# Quantitative proteomic profiling of the extracellular matrix of pancreatic islets during the angiogenic switch and insulinoma progression

**DOI:** 10.1038/srep40495

**Published:** 2017-01-10

**Authors:** Alexandra Naba, Karl R. Clauser, D. R. Mani, Steven A. Carr, Richard O. Hynes

**Affiliations:** 1 David H. Koch Institute for Integrative Cancer Research, Massachusetts Institute of Technology, Cambridge, Massachusetts, U.S.A; 2 Proteomics Platform, Broad Institute of MIT and Harvard, Cambridge, Massachusetts, U.S.A; 3 Howard Hughes Medical Institute, Massachusetts Institute of Technology, Cambridge, Massachusetts, U.S.A

## Abstract

The angiogenic switch, the time at which a tumor becomes vascularized, is a critical step in tumor progression. Indeed, without blood supply, tumors will fail to grow beyond 1 mm^3^ and are unlikely to disseminate. The extracellular matrix (ECM), a major component of the tumor microenvironment, is known to undergo significant changes during angiogenesis and tumor progression. However the extent of these changes remains unknown. In this study, we used quantitative proteomics to profile the composition of the ECM of pancreatic islets in a mouse model of insulinoma characterized by a precisely timed angiogenic switch. Out of the 120 ECM proteins quantified, 35 were detected in significantly different abundance as pancreatic islets progressed from being hyperplastic to angiogenic to insulinomas. Among these, the core ECM proteins, EFEMP1, fibrillin 1, and periostin were found in higher abundance, and decorin, Dmbt1, hemicentin, and Vwa5 in lower abundance. The angiogenic switch being a common feature of solid tumors, we propose that some of the proteins identified represent potential novel anti-angiogenic targets. In addition, we report the characterization of the ECM composition of normal pancreatic islets and propose that this could be of interest for the design of tissue-engineering strategies for treatment of diabetes.

In 2012, the World Health Organization estimated that 14 million people had received a cancer diagnosis and that 8.2 million deaths were due to cancer^1^. These numbers will undoubtedly decrease as we get a better understanding of the molecules and mechanisms involved in the disease and devise strategies to interfere with tumor progression.

The last few decades of research in the field of cancer biology have demonstrated that, in order to progress, tumors not only need to accumulate genetic alterations but also need to be surrounded by a locally permissive microenvironment^2^. In this context, the tumor stroma, and more particularly the extracellular matrix (ECM), appear to be of great interest^3^. The extracellular matrix (ECM) is a complex meshwork of highly cross-linked proteins that controls organ and tissue organization and mechanical properties. In addition, *via* their interactions with cell-surface receptors, or with growth factors, ECM proteins provide biochemical signals that control various cellular processes such as proliferation, survival, and differentiation of both normal and tumor cells^4^,^5^,^6^. ECM deposition is a hallmark of cancers and has been used by pathologists as a poor-prognosis factor, long before the complexity of the ECM was even appreciated^7^.

We have previously reported the development of a pipeline combining proteomic and bioinformatic approaches to characterize the composition of tissue and tumor ECM^8^,^9^. Using this approach, we have shown that there are qualitative changes in the composition of the ECM between primary tumors and the normal tissues from which they originate^10^, primary tumors and derived-metastases^10^, and tumors of differing metastatic potentials^8^,^11^. Importantly, we and other have also demonstrated that the ECM plays causal roles in cancer progression^11^,^12^,^13^. However the ECM is a very dynamic compartment under constant remodeling^14^ and we still do not know how the composition of the tumor extracellular matrix changes as tumors progress.

In this study, we aimed to characterize the changes in ECM composition that occur during tumor progression. To do so, we chose a mouse model of insulinoma, a subtype of pancreatic neuroendocrine tumors^15^, in which the oncogenic SV40 large T antigen (Tag) transgene is under the control of the rat insulin promoter (RIP) and is thus expressed in all β-cells of the pancreatic islets (RIP1-Tag2 model)^16^. This model is characterized by a high penetrance (all mice will develop invasive tumors by 12–14 weeks of age), and a highly reproducible progression timeline: hyperplastic islets appear by 4 weeks of age, a small fraction of hyperplastic islets will switch on an angiogenic program by 7–9 weeks of age, solid tumors emerge at 10 weeks and progress into large and sometime invasive adenomas by 12 to 13 weeks. The angiogenic switch, the time at which a tumor becomes vascularized, is a crucial event required for tumors to grow and disseminate, indeed solid tumors that are not vascularized will not grow beyond 1 mm^3^ 17,18,19, and it is clear that interactions of vascular cells with ECM proteins play key roles in angiogenesis^20^,^21^,^22^. The RIP1-Tag2 insulinoma model has been widely used to identify the molecules and mechanisms controlling tumor angiogenesis, evaluate the relevance of ECM and ECM receptors in tumor progression^23^,^24^,^25^,^26^,^27^, and to test the efficacy of potential therapeutic approaches to inhibit the angiogenic switch or normalize the tumor vasculature, some of which are currently approved for the treatment of cancer patients^18^,^28^,^29^. In a recent study, Langlois and collaborators compared the mRNA expression profile of non-angiogenic and angiogenic RIP1-Tag2 islets and identified 110 ECM genes up-regulated in angiogenic islets, they termed this set the “angiomatrix” signature. They further showed that the angiomatrix signature correlated with poor prognosis of colorectal cancer and glioblastoma patients^30^.

In order to further understand the contribution of the ECM to tumor progression and the angiogenic switch, we utilized quantitative proteomics, based on isobaric peptide labeling (iTRAQ), to profile the extracellular matrix proteomes or “matrisomes” of normal pancreatic islets, hyperplastic islets, angiogenic pancreatic islet, and insulinomas (Fig. 1A). The quantitative comparison of these matrisomes led to the quantification of 120 ECM proteins of which 9 were detected in significantly higher abundance and 26 in significantly lower abundance during insulinoma progression. In addition, we report the detailed characterization of the extracellular matrix of normal pancreatic islets, which we propose could be of interest to scientists interested in regenerative medicine or tissue bioengineering attempting to design materials supporting the regeneration of islets for diabetic patients.

## Materials and Methods

### Animals

B6.D2-Tg(RIP1-Tag2)2Dh (RIP1-Tag2) mice were by obtained from the NCI Mouse Repository (strain number 01XD5)^16^. RIP1-Tag2 mice were fed a diet with adjusted sucrose and cornstarch (Harlan, Madison, WI) and were monitored and handled according to an animal protocol approved by MIT’s Department of Comparative Medicine and Committee on Animal Care.

### Histochemistry

Normal or tumor-bearing pancreatic samples were fixed in 3.8% formaldehyde and embedded in paraffin. Sections were dewaxed and rehydrated following standard procedures. Hematoxylin and eosin and Masson’s trichrome stainings were performed following standard procedures.

### Pancreatic islet isolation

RIP1-Tag2 mice or wild-type (WT) littermates were sacrificed when they reached 6, 9 or 12 weeks of age by CO_2_ inhalation, in conformity with guidelines of MIT’s Department of Comparative Medicine and Committee on Animal Care. Pancreatic islets were isolated as previously described^31^. In brief, 3 mL of a solution of 1 mg/ml of type IV collagenase (Sigma-Aldrich, Saint Louis, MO) reconstituted in Hank’s Balanced Salt Solution (HBSS) were infused via the common bile duct into the pancreas using a 30G1/2 needle. We chose type IV collagenase because of its low general proteolytic activity. The pancreas was then resected and placed in a tube containing an additional 2 mL of collagenase and incubated for 21 minutes at 37 °C with agitation. The digestion was stopped by adding cold HBSS and the pancreas preparation was centrifuged at 290 g. Islet-enriched pancreas extracts were washed twice in HBSS. The pancreas preparation was then filtered through a 70-μm strainer (BD Biosciences, San Jose, CA) and the retained islets collected in a Petri dish. Finally, islets were hand-picked and separated from contaminating exocrine tissue. For WT and hyperplastic islets, this was facilitated by staining the islets with dithizone (Millipore, Temecula, CA)^31^. Angiogenic islets (from 9-week-old mice) were red in appearance due to the increased vasculature and thus did not need to be stained. Insulinomas were hand-picked from the strainer based on their size. To obtain enough material to conduct proteomic analysis using equivalent amount of tissue for each time point, we collected and pooled material from multiple mice. For each of the two replicate series, we collected islets from ~25–35 6-week-old WT or RIP1-Tag2 mice, angiogenic islets from ~15–20 9-week-old RIP1-Tag2 mice, and insulinomas from 3 to 4 12-week-old RIP1-TAg2 mice.

We will further refer to the different stages of the disease progression as follows: hyperplastic islets collected from 6-week old RIP1-Tag2 mice: T6; angiogenic islets collected from 9-week old RIP1-Tag2 mice: T9; insulinoma collected from 12-week old RIP1-Tag2 mice: T12.

### Pancreatic islet decellularization and ECM enrichment

Normal, hyperplastic and angiogenic islets and insulinomas were homogenized with a Bullet Blender (Next Advance, Averill Park, NY) according to the manufacturer’s instructions. Decellularization (removal of intracellular proteins) and concomitant enrichment of ECM proteins were performed using the CNMCS compartmental protein extraction kit (Millipore, Billerica, MA) as previously described^8^,^32^. The effectiveness of the ECM enrichment was monitored by immunoblotting using the following primary antibodies: rabbit anti-collagen I, mouse anti-GAPDH and rabbit anti-histones (Millipore, Billerica, MA), the rabbit anti-actin antibody (serum 14–1) was generated in our laboratory.

### Quantitative proteomic analysis of ECM-enriched samples

ECM-enriched pellets from each time-point were solubilized and digested as previously described^8^,^32^. In brief, ECM-enriched fractions were solubilized in 8 M urea, disulfide bonds were reduced with dithothreitol (10 mM) and alkylated with iodoacetamide (25 mM), and proteins were digested with PNGaseF, Lys-C, and trypsin at 37 °C. Samples were acidified, desalted using 10 mg HLB Oasis Cartridges (Waters Corp., Milford, MA), and eluted with 60% acetonitrile, 0.1% trifluoroacetic acid.

Aliquots of the 4 time points (6-week-old WT, 6-week-old RIP1-Tag2 or T6, 9-week-old RIP1-Tag2 or T9, and 12-week-old RIP1-Tag2 or T12) were labeled with Isobaric Tags for Relative and Absolute Quantitation (iTRAQ) 4-plex reagents. Samples from each time point were combined (~50 μg each time point). Peptide mixes were separated into 64 fractions by reversed-phase chromatography at high pH (basic pH RP) and the fractions were then recombined by pooling every 8^th^ fraction in a step-wise concatenation strategy (see Supplementary Methods). Each pool was analyzed by LC-MS/MS with an Orbitrap Elite mass spectrometer (Thermo Fisher Scientific, San Jose, CA) (see Supplementary Methods for acquisition parameters).

Mass spectra were interpreted with Spectrum Mill (Agilent Technologies, Santa Clara, CA). High-resolution MS/MS spectra were searched against a UniProt database containing mouse reference proteome sequences (including isoforms and excluding fragments), 41,307 entries. The sequences were downloaded from the UniProt web site on October 17, 2014, redundant sequences removed, and a set of common laboratory contaminant proteins (150 sequences) appended. Peptides identified with a false discovery rate estimate of <1.3% were assembled into identified proteins, and annotated as being ECM-derived or not, as previously described^8^,^9^ (see Supplementary Table 1A and 1B).

Relative protein quantitation was evaluated using iTRAQ ratios for the 4 time points (normal, hyperplastic and angiogenic islets and insulinomas). Spectrum Mill used the reporter-ion intensities to calculate the iTRAQ ratios for each peptide spectrum match (PSM). A protein level iTRAQ ratio was calculated as the median of all PSM level ratios contributing to the protein remaining after excluding those PSMs lacking an iTRAQ label, having a negative delta forward-reverse score (estimated false-positive identifications), or having a precursor-ion purity < 50% (MS/MS has significant precursor isolation contamination from co-eluting peptides). To account for differences in ECM protein amount between individual time-point samples within one iTRAQ 4-plex experiment, all iTRAQ time-point ratios were normalized for the ECM population median in the dataset. It is important to note that protein-abundance ratios measured with iTRAQ quantitation can be compressed by a factor of 20–30% due to co-isolation interference and that real effect sizes might be larger than what was measured^33^.

Detailed information on protein identification and quantitation is provided as Supplementary Methods.

The raw mass spectrometry data and the sequence database used for searches have been deposited in the public proteomics repository MassIVE and are accessible at ftp://massive.ucsd.edu/MSV000080124/.

### Statistical analysis

All protein ratios in the protein quantitation tables from Spectrum Mill containing protein-level log2 iTRAQ ratios were normalized by the median protein-level ratio of the subset of matrisome proteins measured for each reporter-ion channel (time point) and provided in Supplementary Table 1C. This normalization accounts for differences in both the total protein content and the extent of ECM enrichment in each of the 4 samples mixed together. Normalized protein-level log2 iTRAQ ratios were used for the statistical analysis. Each time point was treated as an independent group, with statistical significance of a non-zero log-ratio in any group assessed using a moderated F-test based on the ratio of between-group to within-group variability. The F-test allows capture of trends that are not unidirectional, and the moderated version of the test borrows information from all the observed proteins to assess variance of the ratios in a more robust manner. The moderated F-test is implemented in R (R Core Team, 2014) using the limma library^34^. Limma uses an empirical Bayes approach to implement a robust shrinkage strategy for protein-wise variance estimation, thereby enabling the approach to work well with small number of samples^35^. Our analysis is based on a nominal p-value threshold of 0.05 since we tested only 120 proteins for differential regulation. When corrected for multiple testing, the proteins have a Benjamini-Hochberg FDR p-value < 0.16^36^. Scatter plots were generated in R in conjunction with the moderated F-test. For proteins quantified in both replicates and detected in statistically significant different abundance (nominal p-value < 0.05), a heatmap was generated in R with either the individual or averaged protein-level log2 iTRAQ ratios from both experiments and for each time point, using the heatmap function in the NMF library^37^. The columns were hierarchically clustered with complete linkage and Minkowski distance.

## Results and Discussion

### Pancreatic islet isolation and decellularization

**Figure 1:**
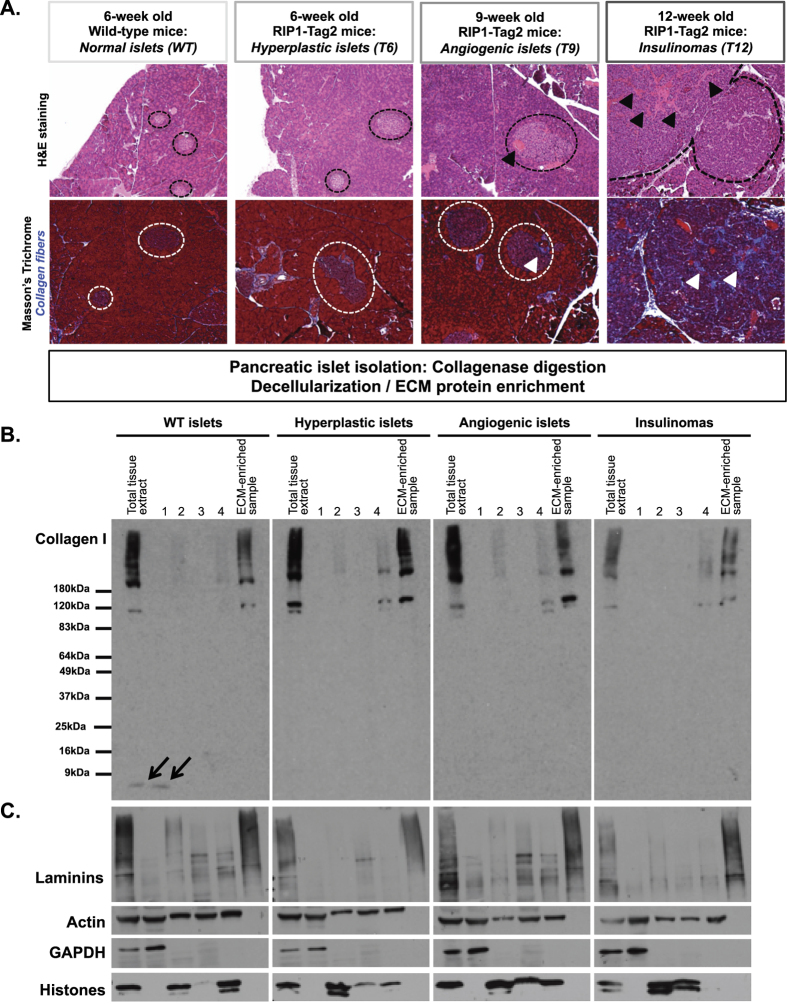
Enrichment of ECM protein from pancreatic islets and insulinomas. (**A**) Hematoxylin and eosin staining (upper panel) or collagen staining using Masson’s trichrome (lower panel) of pancreas from 6-week-old wild-type mice and 6-, 9- or 12-week-old RIP1-Tag2 mice. Dashed lines circle islets and insulinomas. Black arrowheads on the upper panels indicate blood vessels; white arrowheads on the lower panels indicate collagen fibers. (**B**) Anti-collagen I immunoblotting shows that collagen I is not removed during the decellularization of normal and diseased pancreatic islets. Arrows indicate minor soluble fragments of collagen I (<9 kDa) that may be the result of collagenase treatment. (**C**) The sequential extraction of intracellular components during the decellularization process was monitored by immunoblotting for the cytoskeletal protein, actin; the cytosolic protein, GAPDH; the nuclear histones. The insoluble fraction obtained after decellularization was highly enriched for ECM proteins (see collagen I and laminins panels) and largely depleted for intracellular components.

The pancreas comprises an exocrine part composed of acinar cells that produce and secrete digestive enzymes, and an endocrine part, the pancreatic islets, or Langerhans islets, composed of hormone-producing cells including the α-cells producing glucagon and β-cells producing insulin which cooperate to regulate glycemia. Islets are dispersed within the exocrine pancreas and can be distinguished by their histological organization (Fig. 1A). β-cell tumors or insulinomas in the RIP1-Tag2 mouse model progress from hyperplastic islets to angiogenic islets to insulinoma and this progression is accompanied by increased islet vascularization (Fig. 1A, upper panel) and ECM deposition (Fig. 1A, lower panel). In order to profile the composition of the ECM of pancreatic islets during insulinoma progression, we first dissociated the islets from the rest of the pancreas using a mild collagenase treatment. As collagens are major components of the ECM and we are interested in quantifying their abundance in tumors, we monitored by immunoblot that the collagenase treatment did not cause major degradation of the collagens. We could observe only very minor fragments of collagen I (<9 kDa) in the normal islet extracts (Fig. 1B). This loss is minor as the numbers of collagen peptides detected by mass spectrometry are comparable to the numbers of peptides detected in tissues not subjected to collagenase digestion but otherwise processed similarly (Supplementary Table 1A). The decellularization, or removal of intracellular components, of islets was achieved using a method we previously developed that relies on the incubation of tissue extracts in buffers of different salt and detergent composition and takes advantage of the fact that ECM proteins are, by nature, insoluble^8^,^32^. The decellularization resulted (for all four tissues; normal islets, hyperplastic islets, angiogenic islets, and insulinomas) in the enrichment for ECM components (Fig. 1C).

### iTRAQ-based quantitative proteomics of the extracellular matrix of normal pancreatic islets and insulinomas

**Figure 2:**
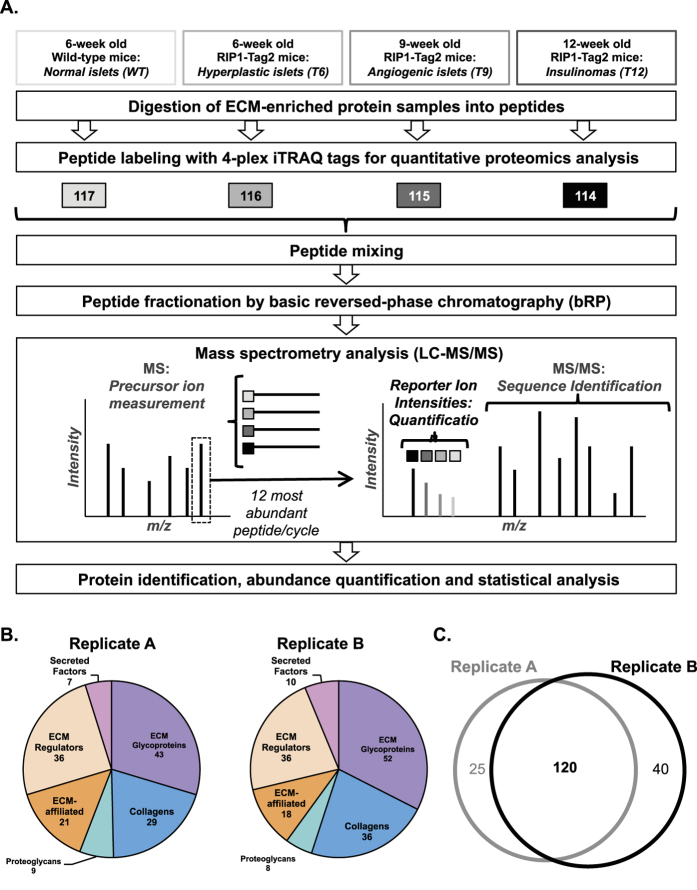
Quantitative proteomic profiling of the ECM of normal pancreatic islets and insulinomas. (**A**) Schematic illustration of the iTRAQ-based quantitative proteomic pipeline used in this study. Fragmentation of MS-selected iTRAQ-tagged peptides in MS/MS yields both peptide identity and relative quantities as it generates low molecular mass-reporter ions with 4 distinct masses (114, 115, 116, and117 - represented by squares). (**B**) Pie charts represent the distribution of ECM and ECM-associated proteins within the different matrisome categories in replicate A (left panel) and B (right panel). (**C**) Venn diagram represents the overlap between ECM and ECM-associated proteins detected in the two biological replicates. The 120 ECM and ECM-associated proteins detected in both replicates are listed in Table 1.

ECM-enriched protein samples obtained from pancreas of wild-type or RIP1-Tag2 mice were digested into peptides. For quantitative proteomic analysis, peptides obtained from normal-pancreatic-islet preparation were labeled using the 117 iTRAQ tag, peptides obtained from islets isolated from RIP1-Tag2 mice at different time points during insulinoma progression (week 6, week 9 and week 12) were labeled with the 116, 115 and 114 tags respectively (Fig. 2A). The four samples were combined, fractionated by basic reversed-phase chromatography and analyzed by tandem mass spectrometry (see Supplementary Methods). Since the four isobaric iTRAQ tags each have the same total mass, the same peptide originating from different samples and labeled with different tags remained indistinguishable during HPLC separation and peptide-precursor-ion-mass measurement by MS. However, the MS/MS fragmentation not only allowed the sequencing of each peptide, but also fragmented the iTRAQ tags to generate low molecular mass reporter ions with 4 distinct masses (114, 115, 116, and117). The reporter-intensity ratios were used for relative quantitation of the four samples for each peptide and eventually the proteins from which they originated (Fig. 2A). The proteomic analysis was conducted on two independent biological replicates and identified 145 ECM and ECM-associated proteins in replicate A and 160 in replicate B (see Supplementary Table 1A and 1B). Furthermore, the distribution of these proteins within the different categories of matrisome proteins was comparable (Fig. 2B).

To further evaluate the quantitative changes in the ECM of pancreatic islets during insulinoma progression, we focused on the 120 ECM proteins quantified in both biological replicates (Fig. 2C). This set of 120 proteins comprises 71 core matrisome proteins (35 ECM glycoproteins, 28 collagen chains, and 8 proteoglycans) and 49 ECM-associated proteins (28 ECM regulators, 15 ECM-affiliated proteins and 6 secreted factors) (Table 1, Supplementary Table 1B). Among the core matrisome proteins detected, a large number are components of basement membranes, including several laminin chains (Lama1, Lama2, Lama3, Lama4, Lama5, Lamb1, Lamb2, Lamc1, Lamc2), nidogens (Nid1 and Nid2), type IV and type VI collagen chains, and heparan-sulfate proteoglycan 2 (HSPG2). We also noted the presence of pancreatic enzymes such as trypsinogens and trypsins (Prss1, Prss2, Prss3, Try4, Try 10) and elastases (Cela1, Cela2a, Cela3b) indicating that, despite enriching for pancreatic islets, our preparation still contained some exocrine pancreatic tissue (see below). This observation might also indicate an increased association of the proteases with insoluble core ECM proteins.

### Identification of changes in the ECM content of pancreatic islets during insulinoma progression

**Figure 3:**
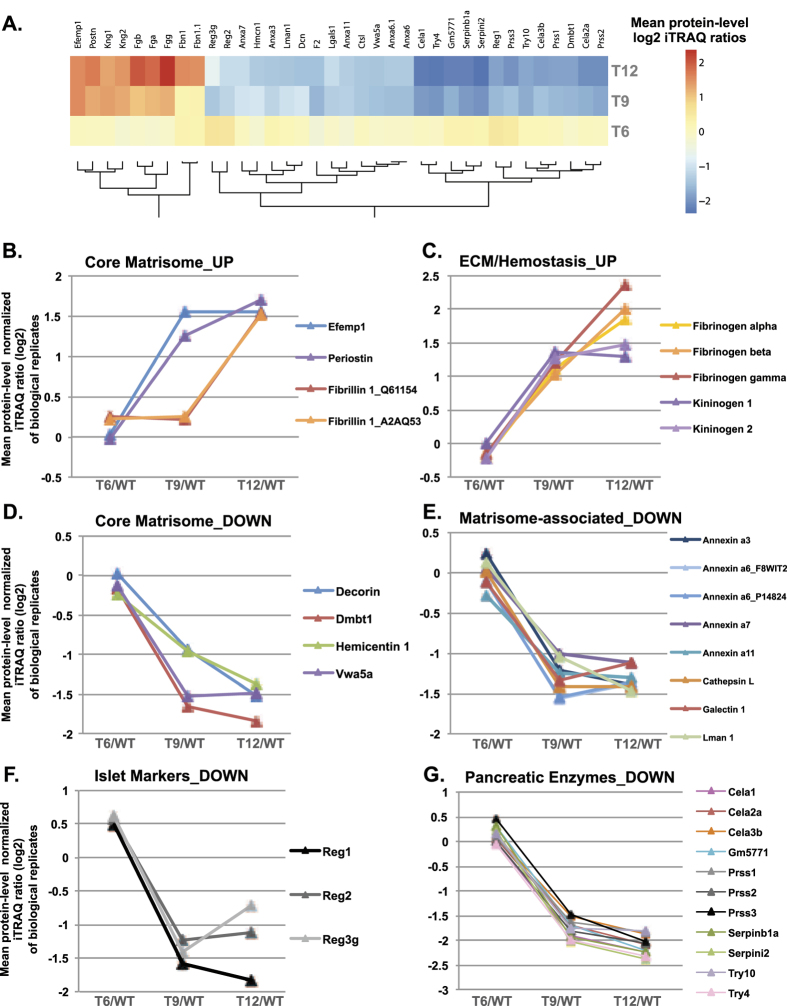
35 ECM and ECM-associated proteins detected in significantly different abundance during insulinoma progression. (**A**) Heat map representing the average protein-level log2 iTRAQ ratios from both experiments for the 36 ECM proteins detected in significantly different abundance (nominal p-value < 0.05) at the different time points of insulinoma progression. (**B–G**) Charts represent proteins showing similar trends during insulinoma progression. Proteins were grouped according to matrisome categories. Levels of detection (mean protein-level log2 iTRAQ ratio) are plotted as a function of tumor progression.

Since our experimental pipeline required pooling of islets from a large number of mice, especially for the early time points (see Materials and Methods section), we evaluated the extent of the reproducibility in protein quantitation (protein-level normalized log2 iTRAQ ratios) in the two biological replicates (Supplementary Figure 1A–C and Supplementary Table 1C, columns F to K). We observed that the abundance of the 120 ECM and ECM-associated proteins quantified in both replicates remains relatively unchanged in hyperplastic islets (isolated from 6-week old RIP1-Tag2 mice) as compared to aged-matched normal islets (Fig. 3A, Supplementary Figure 1D, Supplementary Table 1B). It is also apparent that the data on angiogenic islets (i.e. samples from 9-week-old RIP1-Tag2 mice) time point present more variability than the data from the 6-week-old and 12-week-old RIP-Tag2 samples (Supplementary Figures 1A–C). This may indicate somewhat more advanced insulinoma progression at 9wk in replicate B than in replicate A.

We next sought to identify proteins detected in significantly and consistently different abundance during the course of tumor progression by applying a moderated F-test (see Materials and Methods section, Statistical analysis). Out of the 120 ECM proteins quantified, 35 were detected in statistically significantly different abundance in tumor samples (hyperplastic, angiogenic islets or insulinomas) as compared to normal islets (Fig. 3A, Supplementary Figure 1D, Supplementary Table 1C). Of these, 9 were detected in higher abundance and 26 in lower abundance.

To gain insights into the relevance and implications of this protein signature, we further grouped the proteins based on structural or functional categories (Fig. 3B–G). The first group (Fig. 3B) comprises core matrisome proteins detected in higher abundance as tumors progress. Within this cluster, EGF Containing Fibulin-Like Extracellular Matrix Protein 1 (Efemp1, or fibulin 3) and periostin abundance increased at the angiogenic switch (T9) whereas the abundance of fibrillin 1 only increased in advanced insulinomas (Fig. 3B). In agreement with our results, Efemp1 and periostin have been previously shown to be elevated in various human cancers and to contribute to tumor progression in animal models of cancer, however their functional contribution to the angiogenic switch remains to be demonstrated. Fibrillin 1, however, has not been previously associated functionally with tumor progression. The second group includes ECM and ECM-associated proteins (fibrinogens and kininogens, respectively) involved in hemostasis (Fig. 3C). Their detection in higher abundance may reflect the increased tumor vascularization and the increased leakiness of neo-vessels, as has been observed by others^38^. Note that all 3 fibrinogen chains, that assemble to form a hexamer of two molecules of each chain, follow the same trend.

The 26 proteins detected in significantly lower abundance during insulinoma progression were divided into 4 subgroups. The first subgroup includes 4 core matrisome proteins, decorin, the tumor suppressor Dmbt1 (Deleted In Malignant Brain Tumors 1, also known as muclin), the basement-membrane-associated glycoprotein hemicentin 1 and the von Willebrand factor A domain-containing 5 A (Vwa5a) (Fig. 3D). The proteoglycan decorin has been shown to be anti-angiogenic and to inhibit the progression of different tumor types^39^,^40^. In agreement with this, we observed that the abundance of decorin significantly decreases at the angiogenic switch and is even further decreased in advanced insulinoma. Dmbt1 has been shown to be deleted or mutated in various tumor types^41^,^42^,^43^,^44^. Future work will be required to evaluate whether hemicentin or Vwa5a play functional roles in cancer progression and the angiogenic switch. The second subgroup includes matrisome-associated proteins (Fig. 3E). In addition to several annexins (A3, A6, A7, A11), all significantly decreased in angiogenic islets but not further decreased in insulinoma, we found Lectin, Mannose Binding 1 (Lman1), galectin 1 and the protease cathepsin L to be present in lower abundance in the ECM of both angiogenic islets and insulinomas. Interestingly, the mRNA expression of cathepsin L has previously been shown to be up-regulated in RIP1-Tag2 tumors^45^. This result, contrasting with the proteomic results, suggests that for cathepsin L, the level of mRNA expression is not predictive of the amount of protein found in the insoluble ECM. Further experiments, in particular the evaluation of the level of soluble versus insoluble cathepsin L, are required to decipher the importance of insoluble cathepsin L in insulinoma progression.

Two additional groups of ECM-associated proteins were found in lower abundance during insulinoma progression: regenerating islet-derived proteins (Reg1, Reg2, Reg3g), known markers of normal and regenerating pancreatic islets^46^ (Fig. 3F), and proteins produced by the exocrine pancreas (trypsinogens and trypsins: Prss1, Prss2, Prss3, Try4, Try 10, elastases: Cela1, Cela2a, Cela3b) (Fig. 3G). This observation likely reflects the lower level of contaminating non-islet material, which can be explained by the ease of dissociating encapsulated tumors from the surrounding exocrine pancreas as opposed to the difficulty of purifying normal, smaller islets. It is worth noting that, as the proteomic profiling is conducted on proteins that remained insoluble after tissue decellularization, the detection in higher or lower abundance of certain proteins could not only be due to an increase or decrease in protein production and secretion, but could also be due to an increase or decrease in their solubility.

In a recent study using microarray, Langlois and collaborators compared the mRNA expression profile of angiogenic vs non-angiogenic islets and identified 110 ECM genes up-regulated, they termed this set the “angiomatrix” signature^30^. This study differs form the present study in that it evaluates mRNA level in two oncogenic islet populations (isolated from 8-week-old RIP1-Tag2 mice), angiogenic or not and defined a signature of only up-regulated genes, whereas the present study employs proteomics to evaluate the composition of insoluble ECM during the time-course of tumor progression and uses as reference normal pancreatic islets. Of the core matrisome glycoproteins detected in higher abundance in the present study, two, periostin and fibrillin 1, are part of the angiomatrix signature, suggesting that the increased protein abundance may be due to an up-regulation of the genes encoding them. On the other hand, Efemp1 detected in higher abundance by proteomics is not part of the angiomatrix signature, which can indicate that its increased abundance results in an increase in its insolubility rather than an increase in gene expression. More generally, out of the 38 ECM glycoproteins of the angiomatrix signature, we detected 22 at the protein level, only 2 being significantly more abundant in the present study, and of the 16 glycoproteins not detected in this study, the majority are matricellular proteins (thrombospondin 2 and 4, CCN2 and 4, SPARCL1, IGFBP4 and 5), that are intrinsically more soluble than structural ECM glycoproteins. In addition, we detected all 15 collagens of the angiomatrix signature at the protein level but did not detect any changes in abundance during insulinoma progression. Similarly we detected 7 out of the 9 proteoglycans of the signature. Among those, decorin was found to be up-regulated at the mRNA level, whereas we detected decreased protein abundance. Recent large-scale proteomics studies of colon, breast, and ovarian tumors have reported correlation of mRNA-to-protein with median values of r ~0.4 with significant positive correlation for > 65% of mRNA-protein pairs^47^,^48^,^49^, but these studies did not report correlations focused on ECM proteins. We can postulate that, as the proteomic pipeline employed in the present study focuses on the insoluble ECM, an increase in gene expression could be accompanied by an overall increase in protein abundance but not necessarily by an increased abundance in the insoluble fraction.

### Defining the matrisome of normal pancreatic islets

In humans, insulinoma is a rare subtype of pancreatic neuroendocrine tumors^50^,^51^. They account for approximately 5% of all pancreatic tumors and are malignant in only 10% of the cases. A more prominent disease impacting significantly public health and affecting pancreatic islets is diabetes. In the auto-immune type-1 diabetes, the body does not produce enough insulin to regulate glycemia. Islet transplantation has been proposed to be a longer-term alternative to treatment approaches consisting in insulin injections^52^. Because of the shortage of organs available for transplantation, researchers have turned to regenerative medicine and tissue engineering approaches to generate *in vitro* insulin-producing cells^53^ and in this context, knowing the composition of the *in vivo* islet microenvironment is of particular interest^54^.

We thus sought to exploit the data generated in this study to characterize the ECM of normal pancreatic islets and defined the matrisome of normal pancreatic islets as the ensemble of 120 ECM and ECM-associated proteins detected in both replicates and the additional 58 proteins detected in only one of the two replicates but with at least two unique peptides. According to this definition, the matrisome of normal pancreatic islets is composed of 57 ECM glycoproteins, 37 collagens, 9 proteoglycans, 23 ECM-affiliated proteins, 42 ECM regulators and 10 secreted factors (Table 2, Supplementary Table 1D). Using the combination of precursor- and reporter-ion intensities in the mass spectrometry data (see Supplementary Methods), we were further able to rank the relative abundance of each ECM and ECM-associated protein in normal islets (Table 2, Supplementary Table 1D, column D). Fibrillar collagens, including collagen I, III, and V, fibronectin and fibrillin 1, and components of the basement membranes (such as laminins and nidogens) are the most abundant components of the islet ECM. As compared to the ECM composition of other normal and diseased tissues compiled in an ECM atlas^9^, we have identified 25 proteins (indicated with an asterisk in Table 2) not previously detected in any of the 14 different tissues and tumor types incorporated in the atlas. In addition to known pancreatic- or islet-specific ECM proteins and secreted factors (insulin, elastases, regenerating islet-derived proteins discussed above), we detected for the first time using proteomics, the following structural glycoproteins as components of islet ECM: Sco-spondin (Sspo), Cysteine Rich With EGF Like Domains 2 (Creld2), the proteoglycan Spock2, and the guidance molecule Slit2. Acknowledging the fact that murine and human pancreases present numerous structural and organizational differences^55^, we propose that this first draft of the murine islet matrisome, and in particular the identification of islet-specific ECM proteins, could serve as a guide to engineer environments to support either the differentiation of stem cells into islet cells or the propagation of islet cells *in vitro* for therapeutic purposes.

## Conclusion

Using label-based quantitative proteomics, we report the characterization of the matrisome of pancreatic islets and the identification of a 35-ECM-protein signature characteristic of insulinoma progression. Future work will be required to determine the mechanisms leading to the presence in lower or higher abundance of these ECM proteins in the microenvironment of insulinomas. Indeed the change in abundance of these proteins could result from changes in transcription or translation but could also reflect changes in the secretion, assembly or stability of ECM proteins in the tumor microenvironment. Several of the proteins identified in this study have previously been shown to contribute to tumor progression. In addition, we report here the identification of ECM proteins not previously associated with tumor progression (fibrillin 1, Vwa5a, hemicentin) and it would be interesting to evaluate whether they play a functional role in the angiogenic switch and support tumor progression since, if they do, they could serve as potential anti-angiogenic or anti-tumor therapeutic targets. Finally, we present a comprehensive listing of ECM and ECM-associated proteins in normal pancreatic islets that may be of interest in efforts to develop treatments for diabetes using pancreatic β cells.

## Additional Information

**How to cite this article**: Naba, A. *et al*. Quantitative proteomic profiling of the extracellular matrix of pancreatic islets during the angiogenic switch and insulinoma progression. *Sci. Rep.* **7**, 40495; doi: 10.1038/srep40495 (2017).

**Publisher's note:** Springer Nature remains neutral with regard to jurisdictional claims in published maps and institutional affiliations.

## Supplementary Material

Supplementary Information

Supplementary Table 1

## Figures and Tables

**Table 1:** List of 120 ECM proteins detected and quantified in both replicates.

Core Matrisome		Matrisome-associated	
Glycoproteins	Collagens	ECM Regulators	ECM-affiliated
Dmbt1	Col1a1	Cela1	Anxa1
Dpt	Col1a1	Cela2a	Anxa2
Ecm1	Col1a2	Cela3b	Anxa3
Efemp1	Col2a1	Cst3	Anxa4
Eln	Col3a1	Cstb	Anxa5
Emilin1	Col4a1	Ctsb	Anxa6
Fbln5	Col4a2	Ctsd	Anxa6
Fbn1	Col4a5	Ctsl	Anxa7
Fbn1	Col5a1	F13a1	Anxa11
Fbn2	Col5a2	F2	Lgals1
Fga	Col5a3	Gm5771	Lman1
Fgb	Col6a1	Itih4	Reg1
Fgg	Col6a2	Kng1	Reg2
Fn1	Col6a3	Kng2	Reg3b
Hmcn1	Col6a3	Ngly1	Reg3g
Lama1	Col6a5	Plg	
Lama2	Col6a6	Prss1	**Secreted Factors**
Lama3	Col7a1	Prss2	Hcfc1
Lama4	Col8a1	Prss3	Ins1
Lama5	Col11a1	Pzp	Ins2
Lamb1	Col11a2	Serpina3k	S100a10
Lamb2	Col12a1	Serpinb1a	S100a11
Lamc1	Col12a1	Serpinf2	S100a6
Lamc1	Col14a1	Serpinh1	
Nid1	Col16a1	Serpini2	
Nid2	Col18a1	Tgm2	
Postn	Col27a1	Try10	
Tgfbi	Col28a1	Try4	
Thbs1			
Tinagl1	**Proteoglycans**		
Tnc	Aspn		
Vtn	Bgn		
Vwa1	Dcn		
Vwa5a	Hspg2		
Vwf	Lum		
	Ogn		
	Prelp		
	Prg2		

Proteins are classified as being part of the core matrisome or being matrisome-associated (see Supplementary Table 1B). Within these two divisions, we further annotated core matrisome proteins as being ECM glycoproteins, collagens or proteoglycans, and the matrisome-associated proteins as being ECM-affiliated, ECM regulators or secreted factors.

**Table 2:**
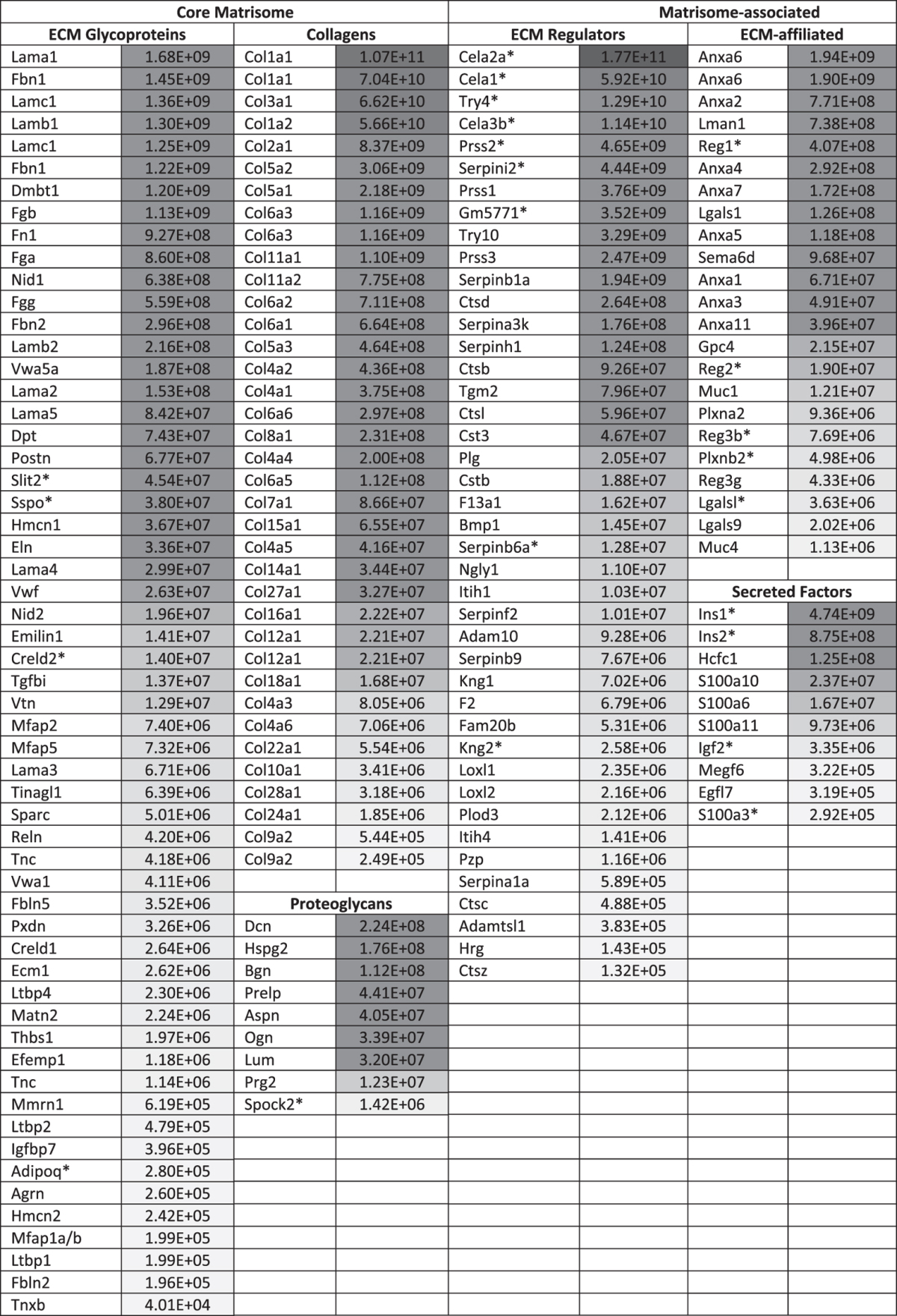
Relative abundance of proteins constituting the matrisome of normal pancreatic islets.

We defined the matrisome of normal pancreatic islets as the ensemble of 120 ECM and ECM-associated proteins detected in both replicates plus the 58 ECM and ECM-associated proteins detected in one of the two replicate but with at least 2 unique peptides.The relative abundances of ECM and ECM-associated proteins in normal islets were calculated as the precursor-ion-weighted average normalized 117-reporter ion intensity (see Supplementary Table 1D).Proteins are sorted by descending relative abundance (Grey scale represents high abundance in dark shades and low abundance in lighter shades).Asterisk (*) indicates proteins not detected in any of the 14 tissues and tumor types contributing to the previously presented ECM atlas^9^.
