# Supplementary material for: Quantitative proteomic profiling of the extracellular matrix of pancreatic islets during the angiogenic switch and insulinoma progression
Source: Sci Rep. 2017 Jan 10;7:40495. doi: 10.1038/srep40495 (PMC5223159; doi:10.1038/srep40495)

Quantitative proteomic profiling of the extracellular matrix of pancreatic islets during the angiogenic switch and insulinoma progression

Alexandra Naba[1#*], Karl R. Clauser[3], D. R. Mani[3]. Steven A. Carr[3], Richard O. Hynes[1,2*]

[1]: David H. Koch Institute for Integrative Cancer Research, Massachusetts Institute of Technology; Cambridge, Massachusetts, U.S.A

[2]: Howard Hughes Medical Institute, Massachusetts Institute of Technology; Cambridge, Massachusetts, U.S.A

[3]: Proteomics Platform, Broad Institute of MIT and Harvard; Cambridge, Massachusetts, U.S.A

#: Present address: Department of Physiology and Biophysics, University of Illinois at Chicago, Chicago, Illinois, U.S.A

* Correspondence:

Alexandra Naba

anaba@uic.edu

Tel: 312-355-5417

Department of Physiology and Biophysics, University of Illinois at Chicago

Room MSB E-202

Chicago, IL 60612, U.S.A.

Richard O. Hynes

rohynes@mit.edu

Tel: 617-253-6422

Koch Institute for Integrative Cancer Research at MIT

Room 76-361

Cambridge, MA 02139, U.S.A.

SUPPLEMENTARY METHODS

iTRAQ labeling

Desalted peptides were labeled with 4-plex iTRAQ reagents as directed by the manufacturer (AB Sciex, Foster City, CA), where 1 unit of labeling reagent was used for each time-point sample. 80 µg dried aliquots of each of the 4 time points (normal, hyperplastic islets, angiogenic islets, and insulinomas) for an experiment were reconstituted in 30 µL 1 M triethylammoniumbicarbonate (TEAB). 70 µL ethanol were added to each sample. 1 unit of iTRAQ reagent (~20 µl) was added to each sample, mixed and incubated at room temperature for 1 hour. Two microliters of each sample were used to check label incorporation by LC-MS/MS prior to quenching the reaction. Unquenched bulk samples were stored at -80°C. After verifying that labeling efficiency was satisfactory (>95% label incorporation), the reactions were quenched by adding 5µl 1M Tris pH 8 for a final concentration of ~50 mM and incubating at room temperature for 15 minutes prior to mixing the samples. For experiment 1 the initial label incorporation was unsatisfactory (70-90%), with the normal and hyperplastic islet samples being the lowest. For re-labeling, the frozen bulk samples were dried down to 30 µL, followed by addition of 70 µL ethanol and another unit of iTRAQ reagent as described above. Labeled samples of 4 different time points were mixed together, dried down and desalted using Oasis HLB 1cc (30mg) reversed-phase cartridges as previously described for post-digestion clean up [1,2]. Eluates were reduced in volume to near dryness and stored at -80°C.

Off-line fractionation of peptides by reversed-phase chromatography at high pH (basic-pH RP)

Desalted 4-plex iTRAQ-labeled peptide mixtures for each experiment were reconstituted in 540 µL of 20 mM ammonium formate/2% acetonitrile pH 10, loaded on a Zorbax 300 Extend 2.1 x 150 mm column (Agilent Technologies, Santa Clara, CA), and fractionated on an Agilent 1100 Series HPLC instrument by basic-reversed-phase chromatography at a flow rate of 200 µL /min. Mobile phase consisted of 20 mM ammonium formate/2% acetonitrile pH 10 (buffer A) and 20 mM ammonium formate 90% acetonitrile pH 10 (buffer B). After loading 500 µL of sample onto the column, the peptides were separated using the following gradient: 5 min. isocratic hold at 0%

B, 0 to 15% solvent B in 8 min.; 15 to 28.5% solvent B in 33 min.; 28.5 to 34% solvent B in 5.5 min.; 34 to 60% solvent B in 13 min., for a total gradient time of 64.5 min. Using 96 x 2mL-well plates (Whatman, #7701-5200) fractions were collected every 0.77 min, 154 μl for a total of 64 fractions through the main elution profile of the separation. The extreme early and late portions of the gradient were collected into two additional larger volume fractions, but not further analyzed. For each experiment all fractions were acidified to a final concentration of 1% formic acid and the fractions were then recombined by pooling every 8th fraction in a step-wise concatenation strategy, as previously reported [3], to yield a total of 8 fractions per experiment. All fractions were dried by vacuum centrifugation and stored at -80°C until mass spectrometric analysis.

NanoLC-MS/MS analysis

Basic-pH RP fractions were reconstituted in 10-15 μl of 3% acetonitrile / 0.1% TFA and 1 μl was analyzed on an Orbitrap Elite mass spectrometer (Thermo Fisher Scientific, Waltham, MA) equipped with a nanoflow ionization source (James A. Hill Instrument Services, Arlington, MA) and coupled to an EASY-nLC 1000 UHPLC system (Proxeon, Thermo Fisher Scientific). Chromatography was performed on a 75 μm ID picofrit column (New Objective, Woburn, MA) packed in house with Reprosil-Pur C18 AQ 1.9 μm beads (Dr. Maisch, GmbH, Entringen, Germany) to a length of 20 cm. Columns were heated to 50°C using column heater sleeves (Phoenix-ST) to prevent overpressuring of columns during UHPLC separation. The LC system, column, and platinum wire to deliver electrospray source voltage were connected via a stainless-steel cross (360μm, IDEX Health & Science, UH-906x). The mobile-phase flow rate was 200nL/min and comprised of 3% acetonitrile/0.1% formic acid (Solvent A) and 90% acetonitrile / 0.1% formic acid (Solvent B). A 124-minute LC-MS/MS method followed a 10-minute column-equilibration procedure and a 6-minute sample-loading procedure for a 1 μL injection. The elution portion of the LC gradient was 0-5% solvent B in 2 min., 5-35% in 90 min, 35-59% in 12 min., 59-90% in 2 min., and held at 90% solvent B for 10 min. to yield ~12 sec. peak widths. Data-dependent LC-MS/MS spectra were acquired in ~2 sec. cycles; each cycle was of the following form: one full Orbitrap MS scan at 60,000 resolution followed by 12 HCD MS/MS scans in the Orbitrap at 15,000 resolution using an isolation width of 2.5 m/z. Dynamic exclusion was enabled with a mass width of +/- 20 ppm, a repeat count of 1, and an exclusion duration of

50 seconds. Charge-state screening was enabled along with monoisotopic precursor selection to prevent triggering of MS/MS on precursor ions with unassigned charge or a charge state of 1. For HCD MS/MS scans the normalized collision energy was 33, AGC target 50,000 ions, and max ion time 200 msec.

Protein identification

All MS data was interpreted using a fully automated workflow in Spectrum Mill software package v6.0 pre-release (Agilent Technologies, Santa Clara, CA). Similar MS/MS spectra acquired on the same precursor m/z within +/- 40 seconds were merged. MS/MS spectra were excluded from searching if they failed the quality filter by not having a sequence tag length > 0 (i.e., minimum of two masses separated by the in-chain mass of an amino acid), did not have a precursor MH+ in the range of 750-6000, or a precursor charge > 5. MS/MS spectra were searched against a UniProt database containing mouse reference proteome sequences (including isoforms and excluding fragments), 41,307 entries. The sequences were downloaded from the UniProt web site on October 17, 2014, redundant sequences removed, and a set of common laboratory contaminant proteins (150 sequences) appended. Search parameters included: ESI-QEXACTIVE-HCD-v2 scoring, parent and fragment mass tolerance of 20ppm, 40% minimum matched peak intensity, trypsin with up to 4 missed cleavages (to allow for Lys-C tendency to cleave at Lys-Pro), and calculate reversed database scores enabled. Fixed modifications were carbamidomethylation at cysteine. To allow for incomplete label incorporation, iTRAQ labeling was required at lysines, but peptide N-termini were allowed to be either labeled or unlabeled. Allowed variable modifications were acetylation of protein N-termini, oxidized methionine, deamidation of asparagine, pyro-glutamic acid at peptide N-terminal glutamine, pyro-carbamidomethylation at peptide N-terminal cysteine, and hydroxylation of proline with a precursor MH+ shift range of -18 to 97 Da. Hydroxyproline was only observed in the proteins known to have it (collagens and proteins containing collagen domains, emilins, etc.) and only within the expected GXPG sequence motifs. The detailed peptide spectral matches might have some examples not in the expected motif when there is either a proline near the motif for which the spectrum could have had insufficient fragmentation to confidently localize the mass change to a particular residue, or a nearby methionine in the peptide and the spectrum had insufficient fragmentation to localize the mass change to oxidized Met or hydroxyproline. When the motif

nX[ST] occurs in a peptide, this is likely to indicate a site where N-linked glycosylation was removed by the PNGaseF treatment of the sample. While a lowercase n indicates a gene-encoded asparagine residue detected in aspartic acid form, possible mechanisms of modification such as acid-catalyzed deamidation during sample processing versus enzymatic conversion during deglycosylation cannot be explicitly distinguished.

Peptide spectrum matches for individual spectra were automatically designated as confidently assigned using the Spectrum Mill autovalidation module to apply target-decoy-based false-discovery rate (FDR) scoring threshold criteria via a two-step auto-threshold strategy at the peptide and protein levels. First, peptide autovalidation was done for each experimental replicate of 8 LC-MS/MS run using an auto-thresholds strategy with a minimum sequence length of 6, automatic variable range precursor mass filtering, and score and delta Rank1 – Rank2 score thresholds optimized to yield a spectral level FDR estimate for precursor charges 2 thru 4 of <1.6% for each precursor charge state in each LC-MS/MS run. For precursor charge 5, thresholds were optimized to yield a spectral level FDR estimate of <0.8% across all 8 runs per experiment (instead of each run), to achieve reasonable statistics since many fewer spectra are generated for the higher charge state. Second, protein polishing autovalidation was applied to further filter all the peptide-level validated spectra with the primary goal of eliminating peptides identified with low scoring peptide spectrum matches (PSMs) that represent proteins identified by a single peptide in a single sample, so-called one-hit wonders. The following parameters were used; minimum number of experiments protein group is observed in: 1, minimum protein score: 15, and maximum protein FDR: 0%. After assembling protein groups from the autovalidated peptides for an experiment, protein polishing determined the maximum protein-level score of a protein group that consists entirely of distinct peptides estimated to be false-positive identifications (PSMs with negative delta forward-reverse scores). Then PSMs were removed from the set obtained in the initial peptide-level autovalidation step if they contribute to protein groups that have protein scores at or below the larger of the minimum protein score and the max false-positive protein score. A protein group would be estimated to be a false-positive if it was identified entirely on the basis of peptides estimated to be false positives. None of these remain after the thresholding in the protein-polishing step. In the filtered results each identified protein was detected with multiple peptides unless a single excellent scoring peptide was the sole match. These autovalidation steps yielded a spectrum level FDR estimate of < 0.7% and a peptide level

FDR estimate of < 1.1% for each experiment. In aggregate across both experiments the estimated FDRs are at the spectrum level: 0.64%, at the peptide level: 1.22%, and at the protein level: <0.03% (1/3701). Since the protein-level FDR estimate neither explicitly requires a minimum number of distinct peptides per protein nor adjusts for the number of possible tryptic peptides per protein, it may underestimate false positive protein identifications for large proteins observed only on the basis of multiple low scoring PSMs.

In calculating scores at the protein level and reporting the identified proteins, redundancy is addressed in the following manner: the protein score is the sum of the scores of distinct peptides. A distinct peptide is the single highest scoring instance of a peptide detected through an MS/MS spectrum. MS/MS spectra for a particular peptide may have been recorded multiple times, (i.e. as different precursor charge states, in adjacent bRP fractions, or different modification states) but are still counted as a single distinct peptide. When a peptide sequence >8 residues long is contained in multiple protein entries in the sequence database, the proteins are grouped together and the highest scoring one and its accession number are reported. In some cases when the protein sequences are grouped in this manner there are distinct peptides which uniquely represent a lower scoring member of the group (isoforms, family members, or different species). Each of these instances spawns a subgroup and multiple subgroups are reported and counted towards the total number of proteins. Peptides shared between subgroups were counted toward each subgroup's count of distinct peptide- and protein-level iTRAQ quantitation. As listed in Supplemental Table 1A, assembly of confidently identified PSMs from both experiments into proteins yields 4135 total protein subgroups from 3701 protein groups.

The raw mass spectrometry data and the sequence database used for searches have been deposited in the public proteomics repository MassIVE and are accessible at ftp://MSV000080124@massive.ucsd.edu.

We further used the matrisome classification we previously defined [4] to categorize all of the identified protein subgroups as being ECM-derived or not (Supplemental Table 1B).

Protein quantitation

Relative protein quantitation was done using iTRAQ ratios for the 4 time points (normal islets, hyperplastic islets, angiogenic islets, and insulinomas). Reporter-ion intensities were corrected for isotopic impurities in the Spectrum Mill protein/peptide summary module using the static

correction method and correction factors obtained from the reagent manufacturer's certificate of analysis for lot number A2157: http://sciex.com/Documents/Downloads/Certificates of Analysis/Certificates of Analysis for iTRAQ Reagents/iTRAQ-Reagent-Multiplex-Kit-4352135-A2157.pdf. Spectrum Mill used the reporter-ion intensities to calculate the iTRAQ ratios for each PSM. A protein-level iTRAQ ratio was calculated as the median of all PSM level ratios contributing to the protein remaining after excluding those PSMs lacking an iTRAQ label, having a negative delta forward-reverse score (half of all false-positive identifications), or having a precursor-ion purity < 50% (MS/MS has significant precursor isolation contamination from co-eluting peptides). To account for differences in ECM protein amount in between single time point samples within one iTRAQ 4-plex experiment, all iTRAQ time-point ratios were normalized for the ECM-population median in the dataset. It is important to note that protein abundance ratios measured with iTRAQ quantitation can be compressed by a factor of 20-30% due to co-isolation interference and that real effect sizes might be larger than what was measured[5].

REFERENCES

1. Naba, A. *et al*. The matrisome: in silico definition and in vivo characterization by proteomics of normal and tumor extracellular matrices. *Mol. Cell. Proteomics* **11,** M111.014647 (2012).
2. Naba, A., Clauser, K. R. & Hynes, R. O. Enrichment of extracellular matrix proteins from tissues and digestion into peptides for mass spectrometry analysis. *J. Vis. Exp.* **101,** e53057 (2015).
3. Wang, Y. *et al*. Reversed-phase chromatography with multiple fraction concatenation strategy for proteome profiling of human MCF10A cells. *Proteomics* **11,** 2019–2026 (2011).
4. Naba, A. *et al*. The extracellular matrix: Tools and insights for the 'omics' era. *Matrix Biol.* **49,** 10–24 (2016).
5. Ow, S. Y. *et al*. iTRAQ underestimation in simple and complex mixtures: 'the good, the bad and the ugly'. *J. Proteome Res.* **8,** 5347–5355 (2009).
6. Smyth, G. K. Linear models and empirical bayes methods for assessing differential expression in microarray experiments. *Stat. Appl. Genet. Mol. Biol.* **3,** Article3 (2004).
7. Ritchie, M. E. *et al*. limma powers differential expression analyses for RNA-sequencing and microarray studies. *Nucleic Acids Res.* gkv007 (2015). doi:10.1093/nar/gkv007
8. Benjamini, Y. & Hochberg, Y. Controlling the False Discovery Rate: A Practical and Powerful Approach to Multiple Testing. *J. R. Stat. Soc. Ser. B Methodol.* **57,** 289–300 (1995).
9. Gaujoux, R. & Seoighe, C. A flexible R package for nonnegative matrix factorization. *BMC Bioinformatics* **11,** 367 (2010).

SUPPLEMENTARY TABLE LEGEND

Supplementary Table 1: Complete MS data set of ECM-enriched preparations.

A. Post-fractionation MS dataset including, for each of the proteins quantified in each replicate, number of spectra (columns D and T), total intensity or peptide abundance (columns E and U), number of unique peptides (columns F and V), reporter-ion intensities (columns G to J and W to Z), and normalized log2 iTRAQ ratios (columns K, N, Q and AA, AD, AG). Proteins are annotated as being part of the matrisome or not (column A) and further classified by matrisome categories (column B).

B. ECM proteins detected and quantified in the two replicates. Table was sorted according to matrisome categories.

C. F-test results were calculated for the 120 ECM and ECM-associated proteins detected and quantified in both replicates. Nominal p-values are given in column O. The table is divided in two groups: proteins detected in significantly different abundance or not. Within each group, proteins are further sorted first by matrisome category and then by ascending p-value.

D. Relative abundance of ECM and ECM-associated proteins in normal islets.

Column D: Average Normalized 117 reporter-ion intensity (precursor-ion-weighted)

Column E: Relative molar abundance [Average Normalized117 reporter-ion intensity (precursor-ion-weighted)/MW]

SUPPLEMENTARY FIGURE LEGEND

Supplementary Figure 1:

A - C. Correlations between replicates A and B for all 120 ECM proteins quantified in both experiments and for each time point. Scatter plots represent protein-level normalized iTRAQ (log2) ratios for experiment A (x-axis), vs. the log ratios for experiment B (y-axis). Red colored points correspond to proteins with p-values < 0.05 in a moderated F test (over all time points). For each time point, the histograms show protein-level normalized iTRAQ (log2) ratios for experiment A (upper left) and B (lower right). Pearson correlation values are indicated in the upper right quadrant for each time point.

D. Heat map representing the protein-level log2 iTRAQ ratios from both experiments for the 36 ECM proteins detected in significantly different abundance (moderated F test p-values <0.05) at the different time points of insulinoma progression.

Supplementary Figure 1

A. **B.** **C.**

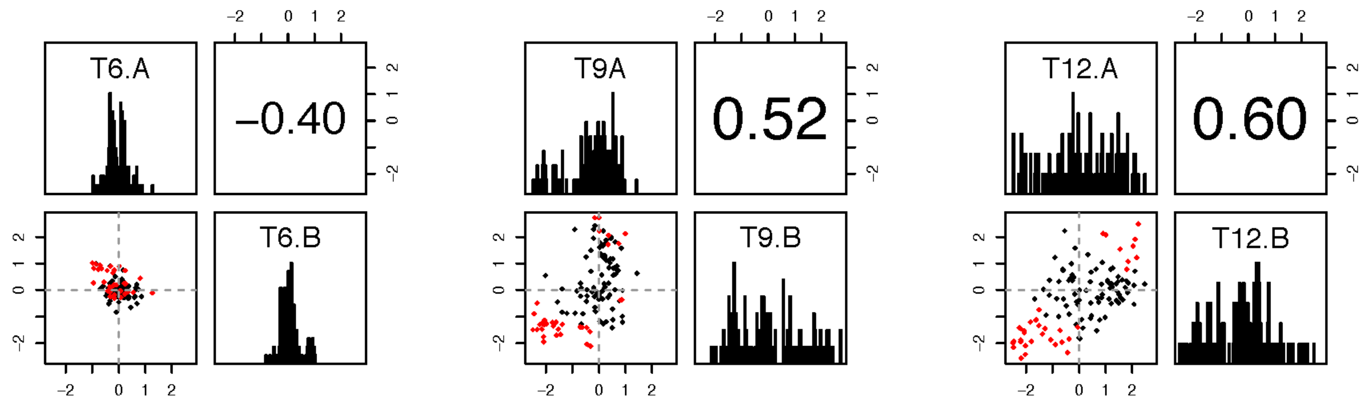

D.

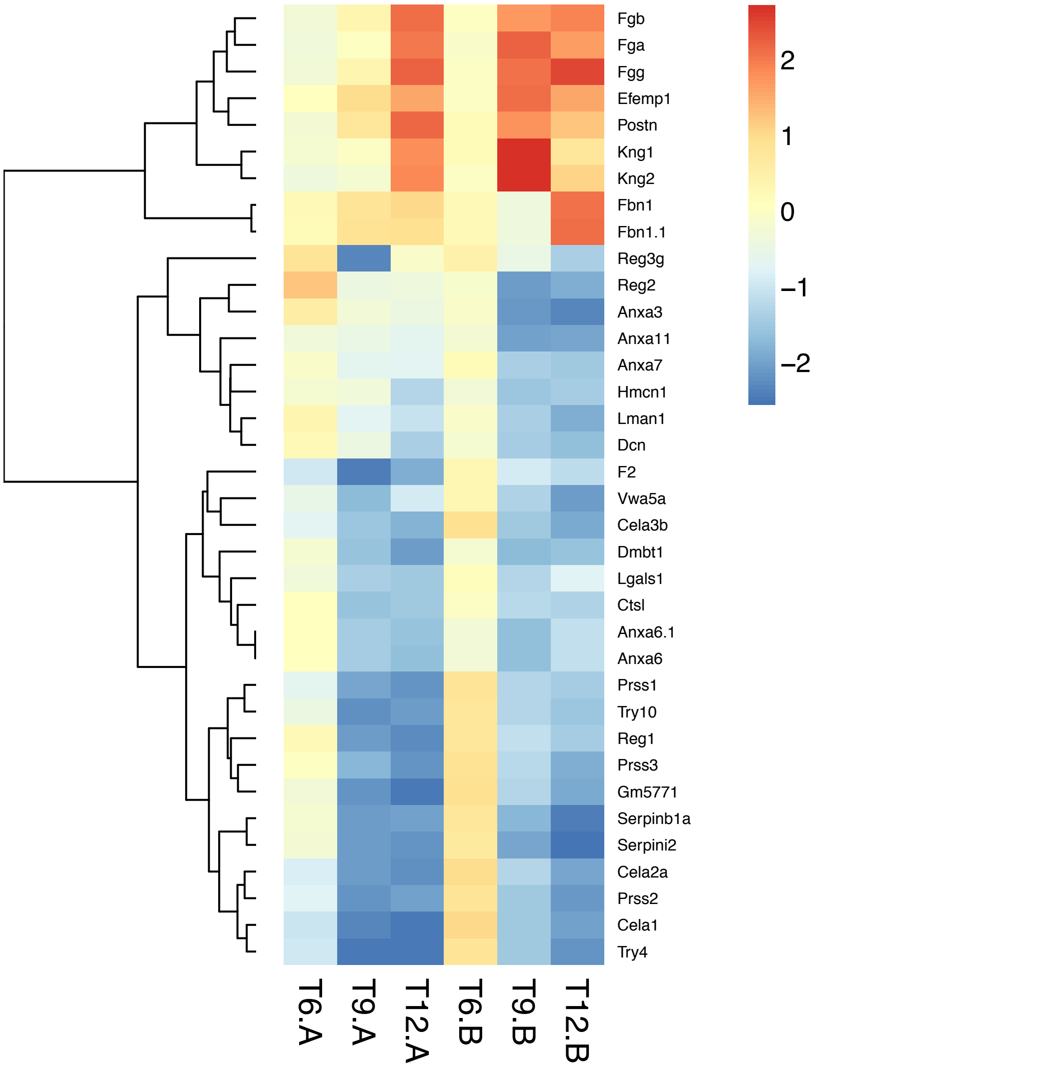

Supplement: Supplementary Information [file srep40495-s1.pdf]
